# Role of the Cingulate Cortex in Dyskinesias-Reduced-Self-Awareness: An fMRI Study on Parkinson’s Disease Patients

**DOI:** 10.3389/fpsyg.2018.01765

**Published:** 2018-09-20

**Authors:** Sara Palermo, Leonardo Lopiano, Rosalba Morese, Maurizio Zibetti, Alberto Romagnolo, Mario Stanziano, Mario Giorgio Rizzone, Giuliano Carlo Geminiani, Maria Consuelo Valentini, Martina Amanzio

**Affiliations:** ^1^Department of Psychology, University of Turin, Turin, Italy; ^2^Department of Neuroscience, University of Turin, Turin, Italy; ^3^Neuroscience Institute of Turin, University of Turin, Turin, Italy; ^4^Faculty of Communication Sciences, Università della Svizzera Italiana, Lugano, Switzerland; ^5^Azienda Ospedaliera Universitaria “Città della Salute e della Scienza di Torino”, Neuroradiology Unit, Turin, Italy; ^6^European Innovation Partnership on Active and Healthy Ageing, Bruxelles, Belgium

**Keywords:** Parkinson’s Disease, dyskinesias, self-awareness, response-inhibition, fMRI

## Abstract

**Objectives:** The detection of dyskinesias-reduced-self-awareness (DRSA), in Parkinson’s disease (PD), was previously associated to executive and metacognitive deficits mainly due to dopaminergic overstimulation of mesocorticolimbic circuits. Response-inhibition dysfunction is often observed in PD. Apart from being engaged in response-inhibition tasks, the anterior cingulate cortex (ACC), is part of a functional system based on self-awareness and engaged across cognitive, affective and behavioural contexts. The purpose of the study was to examine the relationship between response-inhibition disabilities and DRSA using whole-brain event-related functional magnetic resonance imaging (fMRI), over the course of a specific executive task.

**Methods:** Twenty-seven cognitively preserved idiopathic PD patients – presenting motor fluctuations and dyskinesias – were studied. They underwent a neurological and neuropsychological evaluation. The presence of DRSA was assessed using the Dyskinesias Subtracted-Index (DS-I). Cingulate functionality was evaluated with fMRI, while patients performed an ACC-sensitive GO-NoGO task. Association between blood oxygenation level dependent response over the whole-brain during the response-inhibition task and DS-I scores was investigated by regression analysis.

**Results:** The presence of DRSA was associated with reduced functional recruitment in the bilateral ACC, bilateral anterior insular cortex and right dorsolateral prefrontal cortex (*p_FWE_*<0.05). Moreover, DS-I scores significantly correlated with percent errors on the NoGO condition (*r* = 0.491, *p_FWE_* = 0.009).

**Discussion:** These preliminary findings add evidence to the relevant role of executive dysfunctions in DRSA pathogenesis beyond the effects of chronic dopaminergic treatment, with a key leading role played by ACC as part of a functionally impaired response-inhibition network. Imaging biomarkers for DRSA are important to be studied, especially when the neuropsychological assessment seems to be normal.

## Introduction

Dyskinesias are disabling motor complications subsequent to prolonged use of dopaminergic agents in Parkinson’s disease (PD). In particular, levodopa-induced dyskinesia (LID), commonly occurs after long duration of treatment, primarily in the “on-state” medication, negating to some extent its beneficial effects (Vitale et al., 2001; Jenkinson et al., 2009; Amanzio et al., 2010; Sitek et al., 2011).

PD patients may be partially or even completely unaware of the presence of involuntary movements [the so-called *dyskinesias-reduced-self-awareness* (DRSA)]. PD patients with DRSA may therefore not comply with their pharmacological treatment or take part in potentially dangerous activities (Jenkinson et al., 2009). Importantly, DRSA is a clinical phenomenon that can provide untruthful suggestions about the progression of the disease, modify treatment adherence, adversely affect patients quality of life as well as impact the burden of caregivers (Jenkinson et al., 2009).

DRSA has previously been associated with executive dysfunctions due to a dopaminergic overstimulation of mesocorticolimbic circuits (Vitale et al., 2001; Leritz et al., 2004; Amanzio et al., 2010). Indeed, an action monitoring dysfunction, related to the medial prefrontal – ventral striatal circuit including the anterior cingulate cortex (ACC), has been considered to be associated with DRSA, using neuropsychological paradigms (Amanzio et al., 2010, 2014). According to our hypothesis regarding the association between DRSA and executive dysfunction (Amanzio et al., 2010, 2014; Palermo et al., 2017), DRSA can arise when the comparator mechanism for “*attentive-performance-monitoring*” is damaged. In this case, PD patients may not be able to identify their motor symptoms, and dyskinesias do not achieve conscious awareness (Blakemore et al., 2001; Jenkinson et al., 2009). We have therefore demonstrated the harmful role of dopaminergic pharmacological replacement treatment on the prefrontal-subcortical loops producing DRSA, which is linked to specific executive-metacognitive disabilities in terms of global monitoring, monitoring resolution, control sensitivity (Leritz et al., 2004; Amanzio et al., 2010), and the affective component of Theory of Mind (Palermo et al., 2017).

For what is our knowledge, only the study by Maier et al. (2016) analysed neural correlates of impaired self-awareness of motor symptoms in PD. The authors examined this phenomenon in a cohort of twenty-two PD using data from 18F-fluorodeoxyglucose positron emission tomography (Maier et al., 2016), discovering that impaired self-awareness of motor symptoms had its neural substrates in bilateral frontal regions such as the right precentral gyrus, the right superior frontal gyrus, the left inferior frontal gyrus and the medial frontal gyrus (Maier et al., 2016).

Regarding the role of medial prefrontal cortex in impaired self-awareness (Amodio and Frith, 2006), it has been previously demonstrated that a reduced functional recruitment of the MPFC – especially at the level of ACC – may be considered one of the relevant neurobiological substrates of unawareness in early Alzheimer’s disease (Amanzio et al., 2011; Spalletta et al., 2014b), acquired brain injury (Palermo et al., 2014), bipolar disorder (Palermo et al., 2015), and schizophrenia (Orfei et al., 2010; Spalletta et al., 2014a). These results seem to suggest that unawareness of illness in pathologies with different aetiologies may exhibit overlapping symptoms in the context of common patterns of hypofunctionality (i.e., similar neural dysfunction) (Palermo et al., 2014, 2015). Moreover, we have also demonstrated how unawareness is related to the ability to shift and inhibit a response and to action-monitoring (Amanzio et al., 2011; Palermo et al., 2014, 2015), for which ACC functionality is central (Braver et al., 2001; Nee et al., 2007).

As far as we know, no previous studies have evaluated DRSA using an event-related functional MRI (fMRI) paradigm based on response-inhibition. Since the ACC network plays an important role in response-inhibition competence (Braver et al., 2001; Nee et al., 2007), and in our previous study patients who were unaware of their deficits exhibit impaired performance in response-inhibition tasks (Amanzio et al., 2011; Palermo et al., 2014, 2015), we predicted a relationship between DRSA in PD and cingulate hypofunctionality. Considering the above, the two main purposes of the study were: (1) to evaluate possible association between DRSA and response-inhibition performance and (2) to explore and describe the neural substrate of DRSA during a response-inhibition task in PD patients.

## Materials and Methods

### Participants

We prospectively screened 30 patients between January 2016 and November 2017 from the *Parkinson’s and Movement Disorders Unit* of the University of Turin, Italy. Selection was made among patients evaluated for possible access to advanced therapy in terms of deep brain stimulation intervention.

A good clinical response to levodopa with the presence of peak-of-dose dyskinesias and wearing off or on-off phenomena was the first required selection criteria (Amanzio et al., 2010, 2014; Palermo et al., 2017). Subjects took part in the study only if:

(i) they did not have a random on-off
(ii) did not have early-morning and painful dystonia
(iii) they did not show behavioural abnormalities such as major depression, dysthymia or alexithymia based on DSM-V criteria (American Psychiatric Association [APA], 2013)
(iv) they did not have past and present neurological disorder and/or brain organic conditions (other than PD)
(v) they were not taking drug therapies that could directly impact cognitive functioning, other than dopaminergic pharmacological replacement treatment;
(vi) they had more than secondary school education
(vii) they had a Mini Mental State Examination (MMSE) (Folstein et al., 1975) score≥27, in order to include only cognitively non-impaired subjects (Amanzio et al., 2010, 2014; Palermo et al., 2017).

### Procedures

Neurological evaluation was performed both in the absence of drug therapy and over the course of the maximum-benefit-peak of the first daily dose (Amanzio et al., 2010, 2014; Palermo et al., 2017). As regards the off-state, patients were assessed at least 10 h after therapeutic withdrawal, while in the on-state they were assessed within 2 h of the pharmacological assumption. DRSA assessment and the neuropsychological evaluation were performed in the on-state and took at least an hour and a half for each patient.

Importantly, all patients were in therapeutic washout during neuroimaging acquisition, to avoid possible confounding effects of dopamine treatment effects on response-inhibition execution and subsequent fMRI results (Robertson et al., 2015). Indeed, last pharmacological administration was performed in all patients 8 h before the experimental session.

### Neurological Evaluation and DRSA Assessment

The neurological evaluation was performed using the Unified PD Rating Scale revised by the Movement Disorders Society (MDS-UPDRS) (Antonini et al., 2013), which was administered by neurologists blind to the aim of the study. In particular, the motor assessment was performed on the basis of Section III; dyskinesias were assessed using Section IV. Disease stage was rated using the Modified Hoehn and Yahr Scale (Hoehn and Yahr, 1967).

The Dyskinesia rating Scale (DS) (Amanzio et al., 2010, 2014; Palermo et al., 2017) was used to measure awareness of movement disorders. It is a 4-point scale for which the severity of dyskinesias is evaluated separately by the patient and the examiner, while the first performs some selected tasks. Score ranges from 0 (total absence of dyskinesias) to 3 (severe dyskinesias). A *Dyskinesias Index* (DS-I) was calculated by subtracting the patient’s judgments from those of the examiner. Higher scores indicated worse error detection in performance monitoring and so more severe DRSA.

### Neuropsychological and Neuropsychiatric Assessment

The neuropsychological assessment was based on the guidelines by the Task Force commissioned by the Movement Disorders Society to identify Mild Cognitive Decline (Litvan et al., 2012; Goldman et al., 2013). As in our previous studies (Amanzio et al., 2014; Palermo et al., 2017), the test battery included the MMSE to detect the presence of a general cognitive deterioration; attention, perceptual tracking of a sequence and speeded performance were analysed using the Trail Making Test part A (TMT-A); executive functions using the TMT-B and TMT B-A, and the Wisconsin Card Sorting test (WCST); memory abilities with subscales IV and VII of the Wechsler Memory Scale (WMS). Lastly, the ability to access the verbal lexicon was evaluated using the Phonemic Fluency Test – letters F, A, S (FAS) (Amanzio et al., 2014; Palermo et al., 2017).

Neuropsychiatric assessment consisted of the Beck Anxiety Inventory (BAI), the Beck Depression Inventory (BDI), the Apathy Scale (AS), the Young Mania Rating Scale (YMRS) and the Brief Psychiatric Rating Scale 4.0 (BPRS 4.0) (Amanzio et al., 2014; Palermo et al., 2017).

### Scanning Procedure, Activation Paradigm, fMRI Data Preprocessing and Analyses

Neuroimaging data acquisition was performed on a 3T Philips Ingenia scanner (Neuroscience Institute of Turin – Neuroimaging Centre). Images of the whole brain were acquired using a T1-weighted sequence (*TR* = 4.8 ms, *TI* = 1650 ms, *TE* = 331 ms, voxel-size = 1 mm × 1 mm × 1 mm).

During acquisition, the subject was asked to perform a response inhibition ACC-sensitive task (GO-NoGO paradigm), in which the subject had to respond to frequent “GO” stimuli inhibiting the response to infrequent “NoGO” stimuli (the letter “X” with a frequency of 17%), (Braver et al., 2001; Amanzio et al., 2011; Palermo et al., 2014, 2015). Every stimulus was shown for 250 ms with a 1000 ms inter-stimulus interval. The two stimulus types (X and non-X) were presented in random order in a continuous series of 232 trials (Amanzio et al., 2011; Palermo et al., 2014, 2015). Subjects had to respond by pressing a button with their right thumb. The paradigm we used is a prototypical task to measure the ability to inhibit an overpowering response (Braver et al., 2001).

Functional data were acquired using T2^∗^-weighted EPI (*TR* = 2.20 s, *TE* = 35 ms, slice-matrix = 64 × 64, slice gap = 0.28 mm, *FOV* = 24 cm, flip angle = 90°, slices aligned on the AC-PC line).

Image data preprocessing was performed using SPM8, while group-statistics results were visualised using MRIcron. All functional images were spatially realigned to the first volume and anatomical images were co-registered to the mean of them. The functional images were normalised to the Montreal Neurological Institute (MNI), space and smoothed with a 8 mm full-width half-maximum (FWHM), Gaussian Kernel. In order to remove low-frequency drifts, high-pass temporal filtering with a cut-off of 128 s was applied.

After preprocessing, we applied a General Linear Model (GLM) (Friston et al., 2007) to convolve the “GO” and “NoGO” stimuli with canonical hemodynamic response function (HRF). The GLM consisted of two categorical regressors (“GO” and “NoGO” as paradigm conditions), and seven parametric regressors of no interest: six motion regressors in order to correct residual effects of head motion and one (the levodopa equivalent daily dose, LEDD) to exclude any potential influence of pharmacological therapy on fMRI results. At the second level, neural correlates of response-inhibition function were explored by performing a one-sample *t*-test of the contrast “NoGO” vs. “GO” across all the participants. Results were corrected for multiple comparison by small volume correction [SVC], with a sphere of 10 mm radius centred on ACC (our primary region of interest), according to the coordinates reported in the meta-analysis by Need, Wager and Jonides (Nee et al., 2007). Finally, in order to identify which brain regions were associated with DRSA and how task-related activation during response inhibition and DS-I scores were reciprocally correlated, we performed a linear regression of individual scores on DS-I onto whole-brain results for the contrast “NoGO” vs. “GO” (*p_FWE_* < 0.05, at cluster level).

Data from the neuropsychological evaluation are listed in **Table 2**. The neuropsychiatric evaluation showed normative values in both the evaluation phases, on and off. Furthermore, the neuropsychological assessment performed in the on-phase reported normal cognitive profiles.

### Statistical Analysis

Statistical analyses were performed using SPSS version 21.0 (IBM Corp, 2013), for Windows. Data for clinical characteristics and neuropsychological assessment of the subjects are expressed as the mean ± standard deviation.

As far as the “GO-NoGO” paradigm is concerned, patients’ behavioural performance were evaluated in terms of percentage of correct answers (percentage of GO to which the subject responded); percentage of wrong answers (percentage of NoGO to which the subject responded); reaction times (milliseconds from the appearance of the stimulus to the pressure of the response button).

Correlations between DS-I scores and response-inhibition performance were examined using Spearman’s rank-order correlations. A *p*-value of<0.05 was considered statistically significant.

## Results

Of thirty subjects screened, three patients withdrew from the study, while twenty-seven patients (eight women, 19 men), with idiopathic PD, receiving levodopa treatment and presenting motor fluctuations, were enrolled. Disease duration was 10.98 ± 0.94 (mean ± SD) years. The pharmacological treatment had been ongoing for about 8 years and consisted of levodopa associated with dopamine agonists (*LEDD* = 982.86 ± 92.41). Dyskinesias appeared about 3 years before the neuropsychological evaluation. Patients reported normal cognitive profiles at the first level of cognitive profile assessment. Data for key clinical variables are summarised in **Table 1**. More information regarding the experimental sample can be found on **Supplementary Tables I–III**.

**Table 1:** Demographic values and motor assessment.

	Neurological assessment	On-phase (mean ±*SD*)	Off-phase (mean ±*SD*)
Age	mean ±*SD*	64.81 ± 1.31		
Education (years)	mean ±*SD*	9.46 ± 0.98		
Gender (male/female)	19/8			
years since the first symptoms appeared	median [Q1;Q3]	10 [8;13]		
years since the diagnosis was made	median [Q1;Q3]	9.5 [6.75;13]		
years since the dyskinesia appeared	median [Q1;Q3]	3 [1;5]		
hours of Daily *off*	median [Q1;Q3]	2 [0.75;4]		
hours of Daily *on*	median [Q1;Q3]	12 [6.75;14]		
L-dopa (years of treatment)	mean ±*SD*	8.46 ± 0.84		
LEDD (mg)	mean ±*SD*	982.86 ± 92.41		
MDS-UPDRS total score [200]			52.37 ± 4.33	76.59 ± 5.63
Part I [52]			9.50 ± 1.56	10.65 ± 1.63
Part II [52]			10.92 ± 1.20	15.74 ± 1.39
Part III [72]			23.10 ± 2.75	40.91 ± 3.39
Part IV [24]			10.23 ± 1.02	7.67 ± 0.67
Hoehn and Yahr scale [5]			2.12 ± 0.16	2.44 ± 0.12

*Maximum scores for the neurological examination are shown in square brackets. mg, milligrammes; H&Y, Hoehn and Yahr scale; SD, Standard Deviation; Q1, first quartile; Q3, third quartile; N, frequency; MDS-UPDRS, Movement Disorder Society –Unified Parkinson Disease Rating Scale.*

**Table 2:** Neuropsychiatric and neuropsychological assessment in the on-phase of the disease.

		Median	Range	Mean ± Std. Deviation	Cut -off
*Neuropsychiatric assessment*					
AS	[42]	6	2–30	8.28 ± 1.20	≤14
BDI	[39]	6	2–21	7.92 ± 1.01	≤10
BAI	[63]	12	3–23	12.56 ± 1.28	≤21
YMRS	[44]	1	0–21	3-08 ± 1.01	≤12
BPRS 4.0	[168]	38	24–54	37.52 ± 1.59	
*Neuropsychological assessment*					
MMSE	[30]	28	27–30	28 ± 19 ± 0.22	≥24
TMT A	[500]	48	30–159	59.04 ± 5.36	≤94
TMT B	[500]	176.5	70–500	227.23 ± 29.64	≤283
TMT B-A		112.5	29–432	165.35 ± 26.06	≤187
FAS		30	14–50	29.04 ± 1.96	≥17.35
Wechsler Memory Scale 4		7	1.5–19	7.61 ± 0.80	
Wechsler Memory Scale 7		13.5	7–21.5	14.13 ± 0.78	
WCST %		53.12	14–78.13	48.13 ± 3.55	≥37.1
WCST % errors		46.88	21.87–86	52.23 ± 3.54	
WCST % perseverative errors		37.5	14.70–57.14	38.31 ± 2.23	≤42.7
*Reponse Inhibition task: Go*					
Percent Targets	83,72				
Reaction Time		174.68	61.34–396,24	175.66 ± 15.88	
*Reponse Inhibition task: NoGo*					
Percent errors	36.28				
Reaction Time		151.05	69.60–398.32	169.30 ± 17.68	

*Awareness for Levodopa-induced diskinesias is also shown. Where possible, the maximum scores for each test are shown in square brackets. Wherever there is a normative value, the cut-off scores are given in the statistical normal direction; the values refer to the normative data for healthy controls matched according to age and education. Cells in grey indicate the absence of a normative cut-off. N, frequency; AS, Apathy Scale; BDI, Beck Depression Inventory; BAI, Beck Anxiety Inventory; YMRS, Young Mania Rating Scale; BPRS 4.0, Brief Psychiatric Rating Scale version 4.0; MMSE, Mini-Mental state Examination; TMT, Trail Making Test; FAS, Verbal Fluency; WCST, Wisconsin Card Sorting Test; GAM, Global Awareness of Movement Disorders; DS-I, Dyskinesias Subtracted-Index.*

Parkinson’s Disease (PD), patient successfully performed the attentive task (GO) in 84% of cases (correct target), while they properly inhibited the incorrect answer in 64% of cases.

By normal standards, the association between DS-I scores and performance on the GO condition would not be considered statistically significant (*r* = -0.177; *p* = 0.377). Importantly, DS-I scores strongly correlated with percent errors on the NoGO condition (*r* = 0.491, *p* = 0.009). Indeed, the worse the response-inhibition’s performance the worse the ability of a subject to notice and adequately assess the severity of his/her own dyskinesias.

**FIGURE 1:**
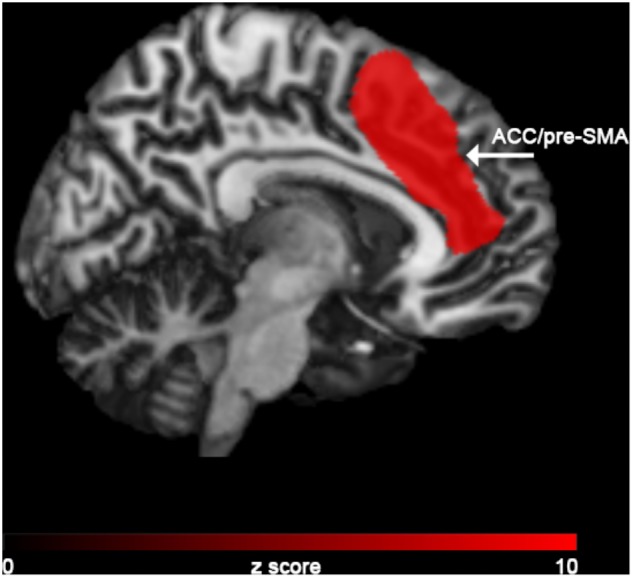
FMRI results from the “NoGO” vs. “GO” contrast.

**FIGURE 2:**
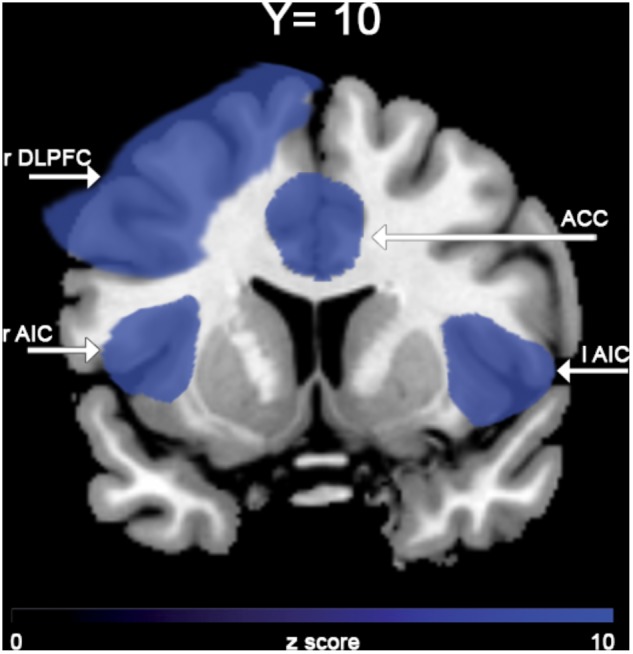
Brain area negatively associated with DS-I scores in the “NoGO” vs. “GO” contrast.

**FIGURE 3:**
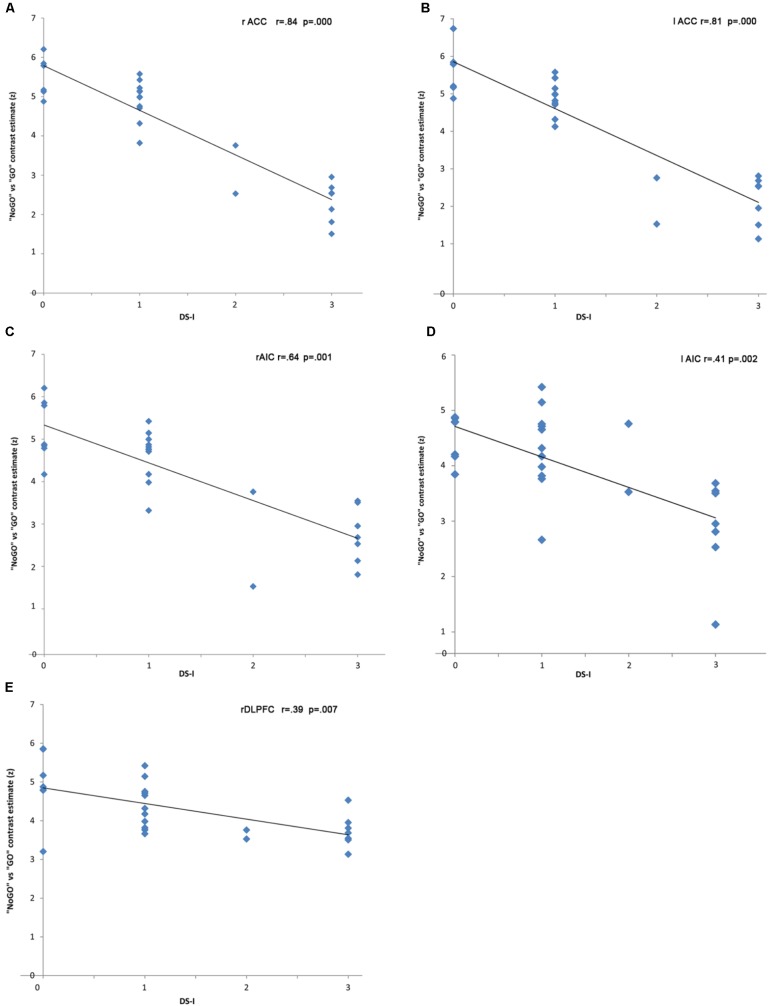
Strength of the fMRI responses (percent change of the BOLD signals) in NoGO trials.

In the “NoGO” vs. “GO” fMRI contrast, expected activation was found in a functional cluster including the bilateral ACC and part of the pre-Supplementary Motor Area (pre-SMA), as shown in **Figure 1**. Linear correlations between neural response during response-inhibition and DRSA scores (as expressed by DS-I) are summarised in **Table 3** and depicted in **Figures 2**, **3**. DS-I scores negatively correlated with the NoGO/GO response in the bilateral ACC, bilateral anterior insular cortex (AIC) and right dorsolateral prefrontal cortex (DLPFC) (*p_FWE_* < 0.05) (see **Table 4**).

**Table 3:** DRSA assessment as measured by the DS-I scale.

Patient code	Patient rate	Clinician rate	DS-I
1	0	3	3
2	2	2	0
3	0	3	3
4	1	2	1
5	1	2	1
6	1	1	0
7	1	2	1
8	2	3	1
9	1	2	1
10	1	2	1
11	0	3	3
12	1	1	0
13	1	1	0
14	1	2	1
15	1	1	0
16	2	2	0
17	1	2	1
18	1	2	1
19	2	3	1
20	1	2	1
21	1	3	2
22	0	3	3
23	0	3	3
24	0	3	3
25	1	3	2
26	0	3	3
27	0	3	3

**Table 4:** Linear correlation between the “NoGO” vs. “GO” contrast and DS-I scores (FWE *p* < 0.05).

	MNI Coordinates			*r*-score	*p*-value
	*X*	*Y*	*Z*		
Brain areas (cluster’s extent in number of voxels)					
rACC (982)	4	26	29	-0.84	0.000
lACC (798)	-5	25	27	-0.81	0.000
rAIC (1855)	33	22	-4	-0.64	0.001
lAIC (1654)	-39	27	3	-0.41	0.002
rDLPFC (2128)	-45	10	47	-0.39	0.007

*Peak activity coordinates are given in MNI space. Peak activities are significant at p < 0.05, FWE corrected for multiple comparisons at the voxel level. ACC, anterior cingulate cortex; AIC, anterior insular cortex; DLPFC, dorsolateral prefrontal cortex; r, right; l, left.*

## Discussion

We studied twenty-seven PD patients to better elucidate the link between brain dysfunction and concomitant cognitive-behavioural disturbances (McGlynn and Schacter, 1989; Lezak et al., 2004; Amanzio et al., 2011; Palermo et al., 2014; Maier et al., 2016), such as DRSA. First, we confirmed the specific and central role of the ACC in the response-inhibition function. Then, we identified a novel negative correlation between DS-I scores and fMRI responses from the NoGO/GO contrast, suggesting that, in our sample, DRSA was specifically associated with reduced functional recruitment of cingulo-frontal (bilateral ACC – R DLPFC), and cingulo-opercular (bilateral ACC – bilateral AIC) regions during the employed response-inhibition task. These findings cannot be merely attributed to cognitive impairment, since all PD patients obtained scores above cut-off on the overall neuropsychological test battery.

It has been reported that DRSA «is characterised by a failure to acknowledge a particular neuropsychological deficit relative to specific functions, i.e., in the case in question, “action”» (Amanzio et al., 2014; Palermo et al., 2017). The phenomenon has attracted growing interest in recent years (Jenkinson et al., 2009; Amanzio et al., 2010, 2014; Maier et al., 2012, 2016; Pietracupa et al., 2013; Palermo et al., 2017). In previous studies approximately half of PD patients exhibited DRSA to some extent (Amanzio et al., 2010; Maier et al., 2012; Pietracupa et al., 2013). In particular, our own studies observed reduced awareness of dyskinesias in 44% (Amanzio et al., 2010), and 53% (Amanzio et al., 2014), of enrolled subjects. Moreover, in the study by Sitek et al., 43% of patients rated their dyskinesias as less severe than did their caregivers (Sitek et al., 2011). More recently, DRSA was reported in 61% and in 23% of PD patients. (Maier et al., 2012, 2016; Pietracupa et al., 2013).

We have demonstrated elsewhere (Amanzio et al., 2010, 2014; Palermo et al., 2017), a significant association between DRSA and reduced functional recruitment of the cingulo-frontal and cingulo-opercular pathways due to prolonged iatrogenic overstimulation and have also already discussed (Amanzio et al., 2010, 2014), possible grounds of inconsistency between our observations and those of others who did not find such an association using neuropsychological approaches (Jenkinson et al., 2009; Maier et al., 2012; Pietracupa et al., 2013; Amanzio et al., 2014). The limitations of these studies, which obtained negative results, had been previously reported (Amanzio et al., 2010, 2014).

This study suggests a possible evidence that chronic dopaminergic overstimulation of mesocorticolimbic circuitries – prolonged over the years – might be considered one of the mechanisms responsible for DRSA pathogenesis. Another possible intervening factor might also go “beyond” chronic dopaminergic overstimulation and affect the mesocorticolimbic circuitries on their own, as we should have ruled out potential confounding effects of replacing pharmacotherapy on our fMRI results: fMRI sequences were acquired in therapeutic washout), and, moreover, LEDD was included as a covariate of no interest (i.e., nuisance regressor), in all individual first level fMRI analyses (in order to control for any residual effect due to “chronic” influence of therapy).

In the case of fMRI contrasts, we only focused on the response-inhibition function as specifically elicited by the “NoGO” vs. “GO” condition and applied regional correction, considering significant results only within the ACC. This was done in order to evaluate, in our sample, the degree of significance in the expected cluster of activation. Against, regression analysis was not restricted to the sole ACC in an effort to not constrain our interpretations to one brain region highly likely to be involved in DRSA pathogenesis. We have here confirmed that ACC is not only significantly active in the contrast NoGO vs. GO, but it is also the area that – among all the areas emerged from the linear regression analysis – expresses the main negative correlation peak with the DS-I scale. We can therefore suggest that ACC could be considered the main hub to interpret DRSA, with the DLPFC and the insula holding the role of supporting actors.

Interestingly, the relationship we found between DRSA and reduced functional recruitment of the cingulo-*frontal* and cingulo-*opercular* pathways refers to regions engaged in loading executive-monitoring onto the processing of task-relevant information, so as to avoid interference by goal-irrelevant stimuli. In particular, the DLPFC is a principal region of the “cognitive-executive” network (CEN), while the ACC and AIC have been identified as major nodes of the “salience” network (SN). These macroscale networks are typically recognised as topographically and functionally distinct from the “default mode” network, which on the contrary subserves inwardly oriented (i.e., self-referential), processing during both wakeful rest and task-execution conditions (Fox et al., 2005; Dosenbach et al., 2007). These areas have to be considered as “hubs” of a wider cognitive control network, globally known as “task positive” (as opposed to the default mode, also called “task negative” network), that includes and connects different functional systems involved in response-inhibition, working memory, action monitoring, representation of the affective qualities of interoceptive signals and sensory events.

The ACC plays important roles in each of these functions (Dosenbach et al., 2006, 2007). Indeed, action monitoring is particularly important in situations that require higher processing capacity. In this case, ACC is concerned with conflict monitoring in several contexts. This includes the online monitoring of responses allowing the identification of errors, as per earlier error-detection theories, and also the detection of conflict between different possible responses to a stimulus, event, or situation. Considering the above, ACC is believed to be involved in attentional processes, particularly “attention for action” (Posner et al., 1988). Most recently, the conflict-monitoring model has been further revised in an effort to consider the findings related to a seeming role for ACC in decision making (Botvinick, 2007). Interestingly the joint action of ACC and AIC can provide an eye-opening perspective on the functions of these regions, as they appear to constitute input (AIC) and output (ACC) hubs of a system based on “awareness of self.” This system may be regarded as an “integrated awareness” of physical, affective and cognitive states, generated by the integrative functions of the AIC and then re-represented in ACC, having the purpose of providing elements for the *selection of*, and *preparation for*, responses to inner or outer events. Our finding of a tight relationship between limited functional recruitment of the cingulo-frontal and cingulo-opercular regions and DRSA suggests that reduction in self-awareness of LID in PD could be interpreted as a specific impairment of an executive function related to metacognitive awareness (i.e., attention-for-action/target selection, motor response selection inhibition and error detection in performance monitoring), in line with our previous results obtained with a neuropsychological approach (Amanzio et al., 2010, 2014; Palermo et al., 2017).

## Limitations Section

The present study has been carefully designed and reached its aims; however, some critical aspects have to be outlined. First, there is no current consensus about standard tools for DRSA assessment, so opting for a scale in place of another could represent a confounding factor. However, DRSA is still detected using different instruments and none of them has been able to prevail as superior to the others. Second, our analyses had been conducted on a relatively small sample, which might reduce statistical power to detect effects and limit generalisation of results. However, our study has to be considered as an exploratory attempt to investigate possible neural underpinnings of DRSA, using an effective and specific ACC-sensitive fMRI paradigm in a selected patient population. Indeed, our sample was clinically homogeneous in terms of disease duration, disease severity, and pharmacological treatment. Moreover, our patients did not present cognitive impairment or behavioural alterations that could compromise the DRSA assessment or the interpretation of the main results.

## Conclusion

This study of DRSA and its neural correlates has relevant clinical implications as this disorder is involved in diagnostic, nosological and prognostic factors that directly affect treatment adherence. Unawareness is often related to poor clinical outcomes and impaired psycho-social functioning. Unaware patients increase caregivers’ burden as they are unable to track changes in their cognitive and behavioural status, thus requiring additional assistance. We believe that theoretical models of unawareness have greater clinical utility and are more effective if they integrate fMRI and neuropsychological data, given the relevance of detecting possible psycho-biological markers of this phenomenon in PD. Importantly, to the best of our knowledge, this is the first study to investigate the relationship between response-inhibition disabilities and DRSA, in cognitively intact patients with PD, using a specific executive (ACC-sensitive) task during an event related fMRI session.

## Future Perspectives

It would be useful to consider the execution of specific response-inhibition task along with the neurological evaluation and neuropsychological assessment in order to define “tailored” interventions in DRSA and adopt a personalised clinical approach avoiding increased doses of dopaminergic drugs, which would in turn enhance the risk of side effects. On the other hand, we have here shown that DRSA pathogenesis in PD may also be considered as “intrinsic” and not necessarily related to chronic dopaminergic overstimulation. Therefore, future studies will be helpful in order to further characterise DRSA features in PD, replicating our findings in a larger group of patients both in the on- and off-phase of daily replacing therapy.

## Ethics Statement

The study was approved by the Ethics Committee “*A.O.U. Città della Salute e della Scienza di Torino - A.O. Ordine Mauriziano - A.S.L. Città di Torino*” as part of the core research criteria followed by the Neurological Units. All the implemented procedures ensured the safety, integrity, and privacy of patients. All subjects gave their informed written consent to participate in the study. Any critical aspects, neither with regard to the fMRI acquisition nor to the neuropsychological assessment could be noticed. Importantly, the study has been conducted according to the principles set forth by the Declaration of Helsinki (59th WMA General Assembly, Seoul, October 2008) and in accordance with the Medical Research Involving Human Subjects Act (WMO).

## Author Contributions

The study was based on a concept developed by MA who wrote the paper and took part in the review and critique processes as PI. SP organised the study, performed the neuropsychological assessment (organisation and execution), participated in the statistical analyses (execution and organisation, review and critique), and wrote the paper. RM and MS conducted the fMRI analyses (execution), and participated in interpretation of results and writing of the paper. MV organised and conducted the MRI acquisition, and participated in interpretation of results and writing of the paper. MZ, AR, and MR performed the neurological assessment (execution) and took part in the organisation of the study and in the diagnostic phase (organisation and diagnosis). GG and LL supervised the neurological evaluation and participated in writing of the paper (review and critique). All the contributors gave their approval of this version of the manuscript to be submitted.

## Conflict of Interest Statement

The authors declare that the research was conducted in the absence of any commercial or financial relationships that could be construed as a potential conflict of interest.
