# Supplementary material for: Role of the Cingulate Cortex in Dyskinesias-Reduced-Self-Awareness: An fMRI Study on Parkinson’s Disease Patients
Source: Front Psychol. 2018 Sep 20;9:1765. doi: 10.3389/fpsyg.2018.01765 (PMC6159748; doi:10.3389/fpsyg.2018.01765)
Supplement: Supplementary file 1 [file Data_Sheet_1.PDF]

	AGE	GENDER	ONSET	DIAGNOSIS (y)	LIDs (y)	DAILY OFF (h)	DAILY ON (y)	UPDRS	UPDRS I	UPDRS II	UPDRS III	UPDR IV	H&Y
1	63	0	10	8	1	2	14	47	3	11	25	8	2
2	74	0	22	19	6	2	13	54	11	10	20	13	2
3	61	0	3	3	2	2	14	59	10	10	26	13	3
4	58	0	10	6	4	2	2	50	5	10	4	27	2
5	72	1	11	8	5	1	11	25	0	3	16	6	2,5
6	70	0	4	4	1	2	15	32	4	9	11	8	2
7	71	1	13	13	4	2	16	108	10	25	57	16	3
8	72	0	11	11	4	5	7	63	30	16	12	5	1
9	63	0	6	4	2	3	16	58	13	9	27	9	3
10	56	0	14	14	3	4	2	49	4	11	27	10	3
11	55	0	10	10	1	4	11			14	5	6	0
12	67	1	8			0	0	65	14	15	25	11	2,5
13	68	0	13	8	3	0	14	24	2	2	13	7	2
14	69	1	10	10	1	2	13	43	4	8	24	13	2,5
15	57	1	9	9	1	2	1	40	6	15	11	8	1,5
16	50	0	3	3	1	0	14	54	7	7	38	6	2
17	66	0	8	7	2	7	9	71	11	12	41	7	2
18	74	1	17	16	7	4	12	16,5	1	0	7,5	23	1
19	70	0	8	7	2	4	9	61	14	14	24	9	3

20	65	1	7	7				19	1	6	8	7	1,5
21	73	0	10	10		0	6	35	1	3	24	7	2
22	64	0	10	10	8	2	5	88	19	24	33	12	2,5
23	60	0	4	4	1	0	12	44	22	4	18	6	1
24	59	0	20	15	2	0	12	66	1	15	45	5	3
25	68	0	14	13	5	9	9	94	19	15	49	11	3
26	69	0	15	15	7	2	13	42	13	16	5	10	2
27	58	1	13	12	5	11	12	54	22	14	10	9	1

Supplementary Table I.

Detailed characteristics of each individual patient. UPDRS and Hoehn and Yahr are given in the on phase. Legend: LIDs= Levodopa-induced dyskinesias; y = years; h = hours; L = left; R = right.

Supplementary Table II.

Characteristics of drug treatment (medication and dosage) of each individual patient.

ID	L-DOPA (y)	First daily dose (mg)	L-DOPA (mg)	DOPAMINE-AGONISTS (mg)
1	7			3,15
2	19	125,00	2335,00	1,05
3	3	125,00	1015,00	3,15
4	6	125,00	1100,00	0,00
5	6	125,00	1175,00	12,00
6	4	100,00	300,00	0,00
7	13	125,00	426,00	2,10
8	11		1050,00	0,00
9	4	275,00	830,00	3,15
10	13	125,00	720,00	10,08
11	7	125,00	1270,00	2,10
12		150,00	965,00	0,00
13	8	175,00	800,00	2,10
14	10	100,00	460,00	3,15
15	9	125,00	901,00	2,10
16	3	250,00	1250,00	3,15
17	7	125,00	705,00	3,15
18	16	125,00	920,00	0,00
19	5			16,00
20	6	125,00	887,80	0,00
21	6	125,00	1221,00	4,00
22	10			0,00
23	4	125,00	1080,00	0,26
24	15	125,00	1290,00	20,00
25	13	250,00	1690,00	1,05
26	10	150,00	865,00	4,20
27	5	125,00	400,00	36,00

Legend: y = years; mg = milligrams.

Supplementary Table III.

The time spent with dyskinesias (MDS-UPDRS 4.1) and the functional impact of dyskinesias (MDS-UPDRS 4.2) for each patient are shown.

ID	MDS-UPDRS 4.1	MDS-UPDRS 4.2
1	3	1
2	3	1
3	4	1
4	2	1
5	2	1
6	1	1
7	4	1
8	4	2
9	2	1
10	3	1
11	1	2
12	2	0
13	2	1
14	2	2
15	2	1
16	2	1
17	2	0
18	1	1
19	3	1
20	3	1
21	1	1
22	1	1
23	4	2
24	2	1
25	1	1
26	2	2
27	1	2
